# Multiparous and primiparous mothers partially differ in how they allocate maternal behaviour in captive western lowland gorillas (*Gorilla gorilla gorilla*)

**DOI:** 10.1007/s10329-025-01195-3

**Published:** 2025-06-06

**Authors:** Federica Amici, Justine Anastasia Pinnecke, Jacques Prieur, Katja Liebal

**Affiliations:** 1 Human Biology and Primate Cognition, Institute of Biology, Faculty of Life Science, University of Leipzig, Leipzig, Germany; 2 Department of Comparative Cultural Psychology, Max Planck Institute for Evolutionary Anthropology, Leipzig, Germany; 3 Comparative Developmental Psychology, Department of Education and Psychology, Freie Universität Berlin, Berlin, Germany

**Keywords:** Parity, Multiparous mothers, Mother-infant relationships, Infant development, Gorillas, Great apes

## Abstract

In primates, mothers are crucial for the survival and integration of young offspring into their social group. Usually, mothers adjust their behaviour to the offspring’s age and needs, but maternal experience might modulate developmental changes in such allocation. In this study, we conducted behavioural observations on 7 mother-offspring dyads of captive western lowland gorillas (*Gorilla gorilla gorilla*) to assess whether multiparous mothers adjusted to the changing requirements of their offspring differently from primiparous mothers, and better facilitated their social integration into the group. Our results showed that, compared to primiparous mothers, multiparous mothers were more likely in body contact with younger offspring and less with older offspring. However, maternal experience neither predicted nor mediated how likely mothers were to start or end body contact, nor did it mediate developmental changes in the offspring’s social behaviour toward other group members. Our study provides preliminary evidence of some limited differences in how primiparous and multiparous mothers allocate maternal behaviour in western lowland gorillas.

## Introduction

In species characterized by slow development, large brains and long lifespans, like primates, young offspring are highly dependent on mothers for their survival (Barton and Capellini 2011; Powell et al. 2019; van Noordwijk 2012). During the first years of their life, primates largely depend on mothers to obtain food, warmth and protection against external dangers (Bales 2017; Brown 2001; Fairbanks 1996; Trivers 1972). Moreover, mothers are crucial to facilitate offspring’s interaction with the physical and social environment, by providing opportunities for social learning that foster the acquisition of new knowledge and skills (Maestripieri 2018; Rosati et al. 2014; Whiten and Waal 2018).

Mothers can also promote offspring’s integration into the social network, through interventions that actively foster relationships with specific partners, and/or through selective passive exposure to them (Berman 1982; de Waal 1990, 1996; Roatti et al. 2023). In this way, mothers can provide offspring with long-term benefits in terms of survival and reproductive success (e.g. Archie et al. 2014; Schülke et al. 2010; Silk et al. 2003, 2009, 2010; but see e.g. Christensen et al. 2024, for evidence that social relationships can also be costly for primates). Indeed, maternal loss during the first years of life is generally associated to high offspring mortality, and to lower survival rate and reproductive success during adulthood (e.g. Crockford et al. 2020; Morrison et al. 2023), although, in some species, social adversity caused by maternal loss might be partially buffered through stronger bonds with other group members (e.g. Morrison et al. 2021).

In primates, mothers are expected to maximize their reproductive success by adjusting their behaviour to the specific requirements of the offspring (e.g. Bercovitch 2002; Clutton-Brock 1991; Trivers and Willard 1973). Through development, for instance, young primates become increasingly able to independently move, feed (i.e. ingest food) and forage (i.e. search for food; e.g. Bründl et al. 2020), and mothers are expected to adjust to these changes by gradually reducing the time spent carrying, nursing and feeding their offspring. These changes can be conflictual, reflecting differences in mothers’ interests (i.e. maximizing reproductive success by investing in multiple offspring) and offspring’s ones (i.e. increasing their own survival chances; Maestripieri 2002; Trivers 1972). As offspring become older, mothers typically become less likely to nurse them and/or maintain proximity to them (e.g. western lowland gorillas, *Gorilla gorilla gorilla*: Maestripieri et al. 2002; Nowell and Fletcher 2007; chimpanzees, *Pan troglodytes*: Pusey 1983; orangutans, *Pongo pygmaeus*: Wich 2009; vervet monkeys, *Chlorocebus pygerythrus*: Fairbanks and McGuire 1987; black-handed spider monkeys, *Ateles geoffroyi*: Sobén López et al. 2023). At the same time, offspring gradually increase the frequency of interactions with other group members, suggesting a progressive integration of immatures into the social network (e.g. chimpanzees: Lonsdorf et al. 2014; macaque spp.: Amici et al. 2019; vervet monkeys: Fairbanks and McGuire 1987).

How mothers allocate maternal behaviour toward offspring might vary depending on their experience. Primiparous mothers (i.e. mothers having given birth to only one offspring), for instance, are usually less experienced than multiparous mothers (i.e. mothers with more than one offspring), they may have longer inter-birth intervals (Gomendio 1989; Jones et al. 2010; Nuñez et al. 2015; Robbins et al. 2006) and give birth to offspring with smaller body size (Altmann and Alberts 2005; Johnson 2003; Setchell et al. 2001) and lower survival rate (Pusey 2012). As a consequence of their inexperience, primiparous mothers might allocate resources to their infants in a different way from multiparous mothers (Bercovitch et al. 2000; Cameron et al. 2000; Fairbanks 1996; Maestripieri 2009; Nguyen et al. 2012). Primiparous mothers, for instance, are often reported to produce less milk and/or milk of lower quality, and to wean their infants later than multiparous mothers (e.g. Japanese macaques: Tanaka 1997; rhesus macaques: Hinde 2009; Hinde et al. 2009; mountain gorillas: Eckardt et al. 2016; chimpanzees: Stanton et al. 2014), possibly to compensate for the lower quality or quantity of their milk (Badescu et al. 2022). With regards to affiliative behaviours, primiparous mothers may be more protective about their infants, spending more time in their proximity and/or approaching them more frequently than multiparous mothers (e.g. cercopithecines: Fairbanks 1996; rhesus macaques: Brown and Dixson 2000; Gomendio 1989; Holley and Simpson 1981; blue monkeys, *Cercopithecus mitis stuhlmanni*: Förster and Cords 2004). Crucially, this might lead infants of primiparous mothers to become independent at a later age, as compared to infants of multiparous mothers (cercopithecines: Fairbanks 1996; Japanese macaques: Bardi and Huffman 2002; Schino et al. 2001).

Here, we aimed to study how maternal experience modulates developmental changes in primate maternal behaviour and, in particular, whether multiparous mothers adjust to the changing requirements of their offspring differently from primiparous mothers, better fostering their social integration into the group. We focused our study on captive western lowland gorillas, a polygynous (Breuer et al. 2009; Robbins and Robbins 2018) and seasonally frugivorous species (Remis 1997; Rogers et al. 2004), in which females usually live in one-male groups with one mature adult male, adult females and immatures (Manguette et al. 2020; Robbins and Robbins 2018). In western lowland gorillas, both sexes usually migrate, but whereas all females migrate from their natal group (usually around 8–9 years of age) and often also show secondary dispersal (Harcourt 1978; Manguette et al. 2020; Robbins et al. 2004, 2016; Stokes et al. 2003), males usually migrate later, having around 18% probability of reaching adulthood (i.e. 18 years of age: Breuer et al. 2009) in the same group of their mother (Robbins et al. 2016).

In western lowland gorillas, mothers are largely responsible for parental care, nursing their offspring until around four and a half years of age (Nowell and Fletcher 2007), although, in closely related mountain gorillas, fathers can also care for their offspring (e.g. providing coalitionary support, feeding tolerance and protection from infanticide; Rosenbaum and Silk 2022; Rosenbaum et al. 2018). In this study, we focused on western lowland gorillas, because previous research in this genus has provided contrasting results with regards to the role of parity on the allocation of maternal behaviour. In mountain gorillas, for instance, multiparous mothers have been reported to show shorter inter-birth intervals than primiparous mothers (Robbins et al. 2006), and wean their offspring earlier, especially females, suggesting an effect of parity on developmental changes in nursing behaviour, and a higher investment of multiparous mothers in sons than daughters (Eckardt et al. 2016). However, other studies have found no significant difference in offspring mortality between primiparous and multiparous mountain gorilla mothers (Robbins et al. 2006), nor in the time western lowland gorilla mothers spend in proximity to their offspring and in how protective they are (Maestripieri et al. 2002). Moreover, given that mountain gorillas and western lowland gorillas face different ecological challenges and are characterized by different social organizations, it may not be possible to generalize findings across species. Furthermore, gorillas may provide important hints about the evolutionary origins of maternal behaviour in humans, given that gorillas share with humans important features of their social organization, like dispersal by both sexes and strong relationships between adult males and females, which are key for reproduction (e.g. Geary and Flinn 2001; Harcourt 1978; Manguette et al. 2020; Robbins et al. 2004, 2016; Stewart and Harcourt 1987; Stokes et al. 2003). Although our sample size was relatively small (*N* = 7), given the difficulty of recruiting more captive mother gorillas with young offspring during the same study period, it allowed us to contribute new data to the study of maternal experience in gorillas, a yet understudied topic.

In this work, we hypothesized that maternal experience would mediate developmental changes in gorilla allocation of maternal behaviour. In particular, we predicted that (1) as compared to primiparous mothers, multiparous mothers would allocate maternal behaviour when mostly needed, providing more body contact and nursing than primiparous mothers to younger offspring, but less to older offspring; (2) multiparous mothers would foster offspring’s social integration into the group more effectively than primiparous mothers.

## Methods

### Study subjects

We conducted behavioural observations on seven mother–offspring dyads of gorillas housed at the Leipzig Zoo, the Burgers’ Zoo and the La Vallée des Singes in France. We included primiparous and multiparous mothers of different age with female or male offspring aged 1 to 40 months at the onset of data collection (for more details, see Table 1). Therefore, we considered the offspring to be dependent infants, as weaning usually occurs after the first 4 years of age in gorillas (e.g. Breuer et al. 2009; Manguette et al. 2020; Nowell and Fletcher 2007). All mothers and offspring were born in captivity, except for a mother, Bebe, who was born in the wild. All study subjects were well habituated to human observers before data collection started.

**Table 1:** For each mother–offspring dyad observed, location, mother’s and offspring’s year of birth and age during the study, offspring’s sex, mother’s parity (P = primiparous, M = multiparous), total number of focal observations and total time of observation

Location	Mother	Offspring	Mother’s birth year	Offspring’s birth year	Mother’s age (years)	Offspring’s age (months)	Offspring’s sex	Parity	Number of focals	Observation time (minutes)
Leipzig Zoo	Viringika	Kibara	1995	2004	8–10	1–18	Female	P	16	401
	Bebe	Louna	1997	2006	27–28	1–15	Female	M	28	1727
Burgers’ Zoo	Shatilla	Shaila	1997	2006	9–10	1–9	Female	P	12	730
	N’Aika	N’Irale	2005	2013	11–12	33–45	Female	P	84	1613
	Makoua	Madiba	2004	2013	12–13	34–47	Male	P	79	1487
	Nimba	Nukta	1999	2013	17–18	40–52	Male	M	88	1593
Vallée des Singes	Moseka	Kouam	1984	2016	32–33	3–15	Male	M	87	1655

All dyads were housed with conspecifics and also with golden-bellied mangabey (*Cercocebus chrysogaster*) at the Burgers’ Zoo and with lesser spot-nosed monkey (*Cercopithecus petaurista*) and mantled guereza (*Colobus guereza*) at the La Vallée des Singes. At the Leipzig Zoo, gorillas were housed in a social group composed of 7 individuals (1 adult male, 2 adult females, 1 subadult male, 2 subadult females, 1 infant). At the Burgers’ Zoo, gorillas were in a social group including 12 individuals (3 adult females, 1 adult male, 2 subadult females, 1 subadult male, 2 infant females, 3 infant males). At the La Vallée des Singes, gorillas were observed in their social group, which was composed of 9 individuals (4 adult females, 1 adult male, 1 subadult male, 1 juvenile female, 1 juvenile male, 1 infant male). All study groups were housed in indoor enclosures with a semi-natural outdoor area of respectively 360 m^2^ and 2300 m^2^ (at the Leipzig Zoo), 125 m^2^ and 3800 m^2^ (at the La Vallée des Singes), 225 m^2^ and 3200 m^2^ (at the Burgers’ Zoo). For a detailed description of the housing conditions at the Burgers’ Zoo and at the La Vallée des Singes, please see Prieur (2015).

### Behavioural observations

Mothers and offspring were video-recorded with a digital video-camera within the official opening hours of the zoo (i.e. from 9 am to 6 pm), using focal animal sampling (Altmann 1974). Given that longitudinal data are difficult to obtain, we combined video footage from different sites that used different observational protocols. Therefore, the length of the focal observations varied from 5 to 60 min, so that multiple sessions were conducted for each dyad to reach a comparable amount of total observation time for most of the dyads included (see Table 1). All dyads were observed during spontaneous interactions in their social group, so that other conspecifics were present in the enclosure and could also be visible in the videos.

We recorded no more than one video a day for each dyad, distributing the observations of each dyad over the course of the day, in all possible contexts (except during feeding times or other human activities that might have affected gorilla behaviour, including the presence of veterinarians around the enclosure and animal presentations, in which keepers introduced the gorillas to the public). In all study groups, observations were made mostly from a slightly elevated area right outside the enclosures, to be as close as possible to the study subjects. At the Leipzig Zoo, several student assistants and interns observed dyads since 2001; for the current study, we included footage from 2004 to 2007, for a total of 103 observation days. At the Burgers’ Zoo, a student assistant observed 1 dyad in 2007 (from February to September), for a total of 15 observation days, whereas J.P. observed the remaining 3 dyads in 2016 (from August to September), for a total of 30 observation days and in 2017 (from July to August), for a total of 31 observation days. At La Vallée des Singes, J.P. observed the dyad in 2016 (from September to October), for a total of 24 observation days and in 2017 (from August to September), for a total of 24 observation days; concerning these 2017 data, we only analysed for the present study footage taken in September, resulting in 18 observation days.

### Coding

We used the software BORIS (v.7.13.6; Friard and Gamba 2016) to code all the videos (*N* = 394). For each video, we entered the identity of the dyad, offspring’s sex and age (in months), mother’s age (in years) and whether the mother was primiparous or multiparous. We then coded (i) the exact time the mother spent in body contact with the offspring (i.e. one mother’s body part was in contact with any body part of the offspring) and (ii) nursing the offspring (i.e. the offspring held the mother’s nipple in the mouth); (iii) the number of times in which the mother and (iv) the offspring started body contact, and (v) the number of times in which the mother and (vi) the offspring ended body contact. We further coded (vii) the exact time the offspring spent in body contact with and (viii) within 2-m proximity to other group members. Finally, we coded the exact duration of the video (excluding the time during which the dyad was not visible, and for 2-m proximity also the time during which the 2-m radius around the infant was not visible), as a measure of observational effort. The occurrence of other affiliative behaviours (e.g. play, grooming) was too low to allow a modelling approach, and their duration was therefore not coded.

### Statistical analyses

We ran six generalized linear mixed models (Baayen et al. 2008) in R (R Core Team 2020), using the package *glmmTMB* (Brooks et al. 2017). For each model, we entered one line for each video coded, so that the dataset of each model had a total of 394 data points. The first four models assessed whether multiparous mothers differed from primiparous ones in how they allocated their maternal behaviour toward offspring during development. In particular, we tested whether the probability of being in body contact with the offspring (Model 1) and nursing the offspring (Model 2) were predicted by the two-way interaction of mother’s experience (i.e. whether the mother was primiparous or multiparous, as a binomial variable) with offspring’s age (in months, *z*-transformed to facilitate model convergence). Both models also included the main terms of the interaction, infant’s sex as control (as mothers might behave differently toward daughters and sons; e.g., Eckardt et al. 2016; Kulik et al. 2016; Murray et al. 2014; Trivers and Willard 1973). The models also included observational effort as offset term and dyad identity nested in group identity as random effect, as observations had different lengths and were not independent from each other. The inclusion of the two-way interaction as predictor allowed us to test whether, compared to primiparous mothers, multiparous mothers provided more body contact and nursing to younger offspring, but less to older offspring (Prediction 1). We opted to model the dependent variables as binomial responses (i.e., whether mothers were in body contact or nursed the offspring during the focal observation), because modelling exact proportions of time as dependent variables led to convergence problems in the models. As a measure of mothers’ experience, we could not include both mothers’ age and parity, as these measures correlated and would have led to high variance inflation factors in the models (Miles 2005). Therefore, we opted to only include parity, as this measure might better capture maternal experience in gorillas (Robbins et al. 2006).

Models 3 and 4 had a similar structure to Models 1 and 2, and they assessed whether the probability that mothers started (Model 3) and ended body contact events (Model 4) was predicted by the two-way interaction of mother’s experience and offspring’s age, as above. As for Models 1 and 2, the inclusion of this interaction allowed testing Prediction 1. Responses were modelled with a binomial structure, using the cbind function (as the number of body contact events started/ended by mothers versus the number of body contact events started/ended by offspring). Therefore, in the models it was not necessary to control for observational effort. However, we further included dyad identity nested in group identity as random effect and the main terms of the two-way interaction, as above.

Finally, we tested whether the probability of being in body contact with (Model 5) and in 2-m proximity to (Model 6) group members other than the mother was predicted by the two-way interaction of mother’s experience and offspring’s age, including observational effort as offset term, dyad identity nested in group identity as random effect and the main terms of the two-way interaction, as above. The inclusion of the interaction allowed us to test whether, compared to primiparous mothers, multiparous ones better fostered social integration into the group, when accounting for infant’s age (Prediction 2). Given that proximity to other group members could simply be a by-product of the presence of mothers, in the models we also included as a control whether mothers were in body contact with the offspring during the focal observation. Moreover, given that proximity to other group members might simply reflect the availability of age-peers in the group, in the models we also included as test predictor the number of infants that were present in each study group; as including this predictor caused overdispersion in Model 6 and did not alter the results compared to models without it (i.e., the same test predictors were significant), we report below the results from the models excluding the number of infants in the study group.

All full models described above were compared with likelihood ratio tests to a corresponding null model, which was identical but did not include test predictors. We set the significance level (α) at 0.05. In case of significant difference between the full and the null model, we used the drop1 function to assess which test predictors were significant. When the interaction was not significant, we re-ran the analyses only including the main terms. We used the performance package (Lüdecke et al. 2021) and the *Dharma* package (Hartig 2022) and did not detect any issues in the models presented, with regards to convergence, distribution of residuals and multicollinearity (maximum variance inflation factors across models after removing the interactions = 2.15; Miles 2005). We refrained from conducting more complex models (e.g. with the three-way interaction of mother’s experience, offspring’s age and offspring’s sex), because of the small sample size (e.g., we only had one male infant with a primiparous mother and a female infant with a multiparous mother).

## Results

**Fig. 1:**
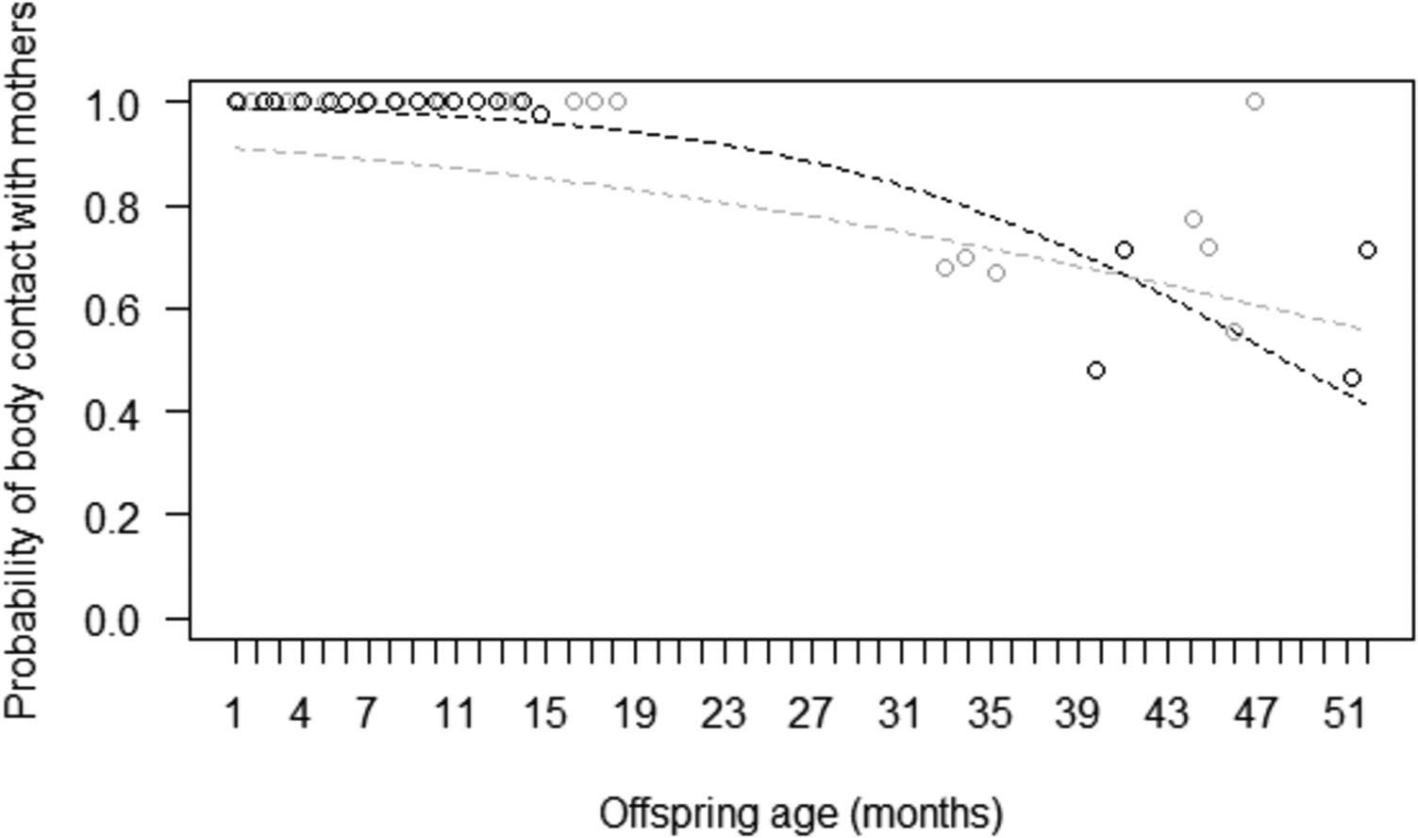
For primiparous and multiparous mothers separately, probability of being in body contact with their offspring, as a function of offspring’s age (in months, slightly jittered to increase visual clarity). Circles represent the mean probability of body contact at each age, separately for primiparous (in grey) and multiparous (in black) mothers, after aggregating the data points used for Model 1. The two lines represent the fitted model, which is like Model 1, but unconditional on the other predictors that were standardized, and with observational effort expressed in 15-min intervals. The probability of being in body contact with their offspring was higher for multiparous mothers during the first months, and then decreased through time for all mothers, but more quickly for multiparous than primiparous mothers

We found variation in the probability of mothers being in body contact with the offspring (Model 1), as the full model significantly differed from the corresponding null model (Table 2). In particular, the probability of body contact varied through development depending on maternal experience (*p* ≤ 0.05): it was higher for multiparous mothers during the first months of offspring’s life, and it decreased through time for all mothers, but more quickly for multiparous than primiparous ones (Fig. 1). Therefore, in line with the first prediction, multiparous mothers appeared more likely to allocate maternal behaviour when mostly needed (i.e. toward younger offspring), as compared to primiparous ones.

**Table 2:** Results of the five models run, including estimates, standard errors (SE), confidence intervals (CIs), likelihood ratio tests (LRT), degrees of freedom (df), and *p* values for all test predictors and controls (in italics); reference categories are in parentheses

Models	Estimate	SE	2.5–97.5% CIs	LRT	*df*	*P*
Model 1: Probability that mothers are in body contact with their offspring (GLMM, *χ*^2^ = 9.05, *df* = 3, *p* = 0.029)						
Intercept	− 5.69	0.28	− 6.23 – − 5.15	–	–	–
Maternal experience * offspring’s age	− 0.92	0.47	− 1.84 – 0.00	3.73	1	0.053
Maternal experience	0.63	0.46	− 0.27 – 1.54			
Offspring’s age	− 0.69	0.36	− 1.40 – 0.01			
Offspring’s sex (male)	0.04	0.36	− 0.66 – 0.74	0.01	1	0.906
Model 2: Probability that mothers nurse their offspring (GLMM, *χ*^2^ = 8.51, *df* = 3, *p* = 0.037)						
Intercept	− 7.86	0.19	− 8.46 – − 7.62	–	–	–
Maternal experience	− 1.31	0.29	− 1.88 – − 0.73	6.43	1	0.011
Offspring’s age	−0.30	0.14	− 0.75 – 0.20	3.65	1	0.056
Offspring’s sex (male)	0.80	0.27	0.26 – 1.36	3.81	1	0.051
Model 3: Probability that mothers start body contact with their offspring (GLMM, *χ*^2^ = 6.67, *df* = 3, *p* = 0.083)						
Intercept	− 1.09	0.19	− 1.47 – − 0.71	–	–	–
Maternal experience	0.23	0.28	− 0.32 – 0.77	0.62	1	0.431
Offspring’s age	− 0.19	0.15	− 0.49 – 0.10	1.92	1	0.166
Offspring’s sex (male)	− 0.06	0.30	− 0.65 – 0.53	0.04	1	0.836
Model 4: Probability that mothers end body contact with their offspring (GLMM, *χ*^2^ = 10.97, *df* = 3, *p* = 0.012)						
Intercept	− 0.79	0.22	−1.22 – − 0.37	–	–	–
Maternal experience	0.16	0.33	−0.48 – 0.80	0.21	1	0.650
Offspring’s age	1.03	0.19	0.66 – 1.41	9.46	1	0.002
Offspring’s sex (male)	− 0.24	0.37	−0.96 – 0.48	0.34	1	0.559
Model 5: Probability that the offspring is in body contact with others (GLMM, *χ*^2^ = 13.52, *df* = 3, *p* = 0.004)						
Intercept	− 2.42	0.84	− 4.06 – − 0.78	–	–	–
Maternal experience	− 1.62	1.20	− 3.97 – 0.73	1.64	1	0.200
Offspring’s age	1.20	0.41	0.40 – 2.01	8.34	1	0.004
Offspring’s sex (male)	2.91	1.24	0.49 – 5.33	4.12	1	0.042
Mother is in body contact	1.96	0.49	1.00 – 2.91	18.01	1	< 0.001
Model 6: Probability that the offspring is in 2-m proximity to others (GLMM, *χ*^2^ = 20.37, *df* = 3, *p* < 0.001)						
Intercept	− 6.58	0.20	− 6.98 – −6.18	–	–	–
Maternal experience	− 0.02	0.29	− 0.59 – 0.55	0.00	1	0.949
Offspring’s age	0.80	0.14	0.52 – 1.07	17.00	1	< 0.001
Offspring’s sex (male)	0.02	0.29	− 0.55 – 0.59	0.00	1	0.947
Mother is in body contact	1.35	0.37	0.63 – 2.07	15.13	1	< 0.001

**Fig. 2:**
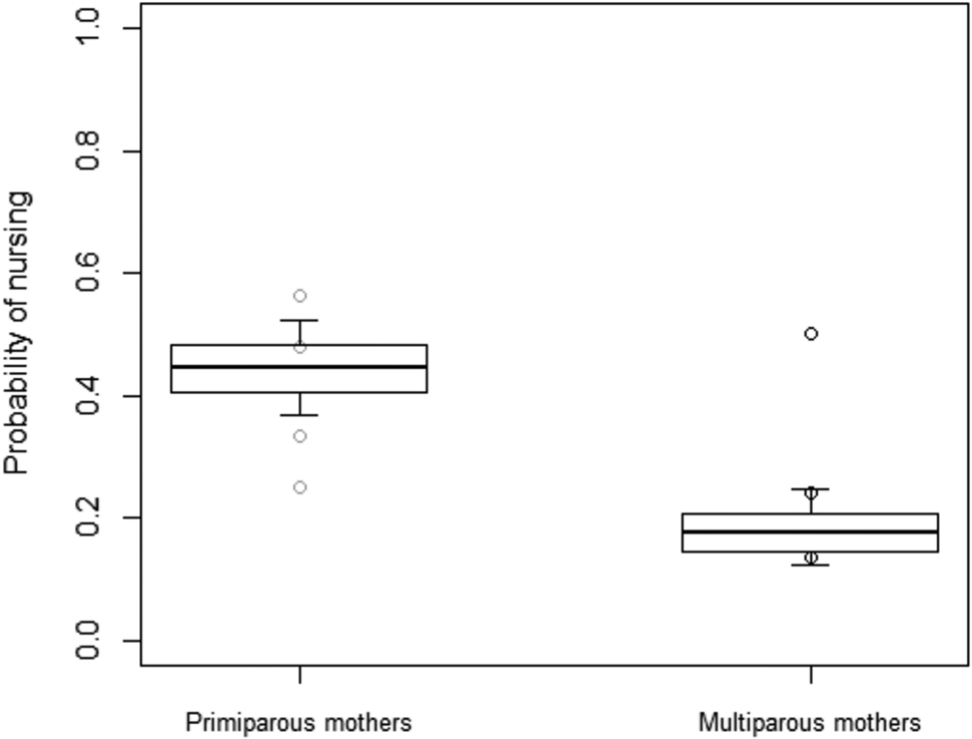
Probability of mothers nursing their offspring, as a function of mother’s experience. Circles represent the mean probability of nursing for each infant, separately for primiparous (in grey) and multiparous (in black) mothers, after aggregating the data points used for Model 2, with observational effort expressed in 60-min intervals. The probability of nursing their offspring was overall higher for primiparous than for multiparous mothers

We further found variation in the probability of mothers nursing their offspring (Model 2), as the full-null model comparison was significant (Table 2). In particular, the probability of mothers nursing offspring was overall higher for primiparous than nulliparous mothers (*p* ≤ 0.05; Fig. 2). Moreover, it tended to decrease through age (*p* = 0.056), and it was overall higher for male than female infants (*p* ≤ 0.05), only partially in line with our first prediction that multiparous mothers would differ from primiparous ones in their allocation of maternal behaviour.

We then tested variation in the probability that mothers would start body contact with their offspring (Model 3), but the full model did not significantly differ from the corresponding null one (Table 2). However, the full-null model comparison was significant when testing variation in the probability that mothers would end body contact with their offspring (Model 4; Table 2). In particular, mothers became relatively more likely than infants to end body contact, as offspring became older (*p* ≤ 0.05). However, mother experience had no significant effect on our response, in contrast to our first prediction.

Finally, the probability of infants being in body contact (Model 5) or proximity (Model 6) to group members other than their mothers did not vary depending on mother’s experience. For body contact, the full model was significantly better than the null one (Table 2): infants became more likely to be with others as they became older (*p* ≤ 0.05), but mother experience had no significant effect on this probability, in contrast to our first prediction. Moreover, the probability of being in body contact with others was higher for male than for female infants, and it increased when mothers were also close to the infants (both *p* ≤ 0.05). The pattern was similar for proximity: the full model was significantly better than the null one (Table 2), infants became more likely to be with others as they became older (*p* ≤ 0.05), but mother experience had no significant effect on this probability, in contrast to our first prediction. Moreover, the probability of being in proximity to others was higher when mothers were also close to the infants (*p* ≤ 0.05).

## Discussion

In our study, all gorilla mothers adjusted to the changing needs of their offspring, and maternal experience had only a very limited modulating effect on these developmental changes. In line with our first prediction, multiparous mothers were quicker than primiparous mothers to decrease body contact with their offspring throughout development (Model 1). Moreover, the probability of nursing offspring was generally lower for multiparous than primiparous mothers, although there were no differences in their developmental trajectories (Model 2). In contrast to our first prediction, maternal experience did not predict the probability of starting (Model 3) or ending (Model 4) body contact with the offspring. Finally, in contrast to our second prediction, multiparous mothers did not appear to better foster offspring’s integration into the social group, as the probability of being in body contact (Model 5) or in proximity (Model 6) to other group members changed throughout the infants’ development, independently of mothers’ experience.

As predicted, multiparous mothers seemed to better adjust to the changing needs of their offspring, as compared to primiparous mothers, by better timing the allocation of body contact when it was likely most needed (i.e. when offspring was younger). In particular, in line with literature in gorillas and other primate species (e.g. Western lowland gorillas: Maestripieri et al. 2002; Nowell and Fletcher 2007; black-handed spider monkeys: Sobén López et al. 2023), all mothers became less likely to nurse and to be in body contact with their offspring, as the offspring became older. Crucially, however, changes in the allocation of body contact were stronger for multiparous mothers: early in life, primiparous mothers generally had less body contact with younger offspring than multiparous mothers, but their probability of being in body contact was more consistent across infancy than for multiparous mothers. These data are in line with research in other species, which suggests that multiparous mothers may allocate their time more effectively than primiparous mothers (rhesus macaques: Bercovitch et al. 2000; cercopithecines: Fairbanks 1996; yellow baboons: Nguyen et al. 2012; horses, *Equus ferus caballus*: Cameron et al. 2000).

With regards to nursing, however, the pattern was different, as multiparous mothers were generally less likely than primiparous mothers to nurse their offspring, regardless of infants’ age. Previous studies have shown that primiparous mothers usually produce milk that has lower quality and/or is less in quantity (e.g. Eckardt et al. 2016; Hinde 2009; Hinde et al. 2009; Stanton et al. 2014; Tanaka 1997), so that mothers have to nurse longer to compensate for this (Badescu et al. 2022; Hinde and Milligan 2011). Possibly, the differences in nursing that we evidenced in our study reflect the fact that primiparous mothers have less and/or lower quality milk, and they thus need to nurse their offspring for longer periods of time, as compared to multiparous mothers.

In contrast to our predictions, maternal experience neither predicted nor mediated how likely mothers were to start or end body contact with their offspring, nor did it mediate developmental changes in the offspring’s social behaviour toward other group members. In contrast, these behaviours simply changed through time, as infants grew up. In particular, regardless of their experience, mothers became gradually more likely to end body contact with their offspring. These findings are in line with literature on primates showing a general decrease in maternal behaviours through offspring’s development (Fairbanks and McGuire 1987; Maestripieri et al. 2002; Nowell and Fletcher 2007; Pusey 1983; Sobén López et al. 2023; Wich 2009), but they fail to confirm our prediction that maternal experience might modulate this pattern. In the same line, the probability of being in body contact or in proximity to other group members increased through infants’ development, but mothers’ experience did not appear to foster this process, in contrast to our second prediction. These findings, and especially the fact that mother’s presence also increased infants’ probability of being close to other group members, align with abundant literature highlighting the crucial role that mothers play for the integration of immatures into the social network (Amici et al. 2019; Fairbanks and McGuire 1987; Lonsdorf et al. 2014). Yet, this important role appears to be played by all mothers, regardless of their experience. Clearly, it is likely that the specific composition of the group may also affect infants’ probability of being in body contact or in proximity to other group members. In our study, however, accounting for the number of age-peers in each group did not alter the results (Models 5–6), suggesting that our results cannot simply be explained by the different number of infants in each study group. In the future, it will be important to better account for the different partners potentially available in the group.

In this study, we operationalized maternal experience as parity, although it is likely that maternal experience encompasses further multiple dimensions, including mothers’ age and previous life experiences. For instance, primiparous mothers are usually younger and might be lower ranking than multiparous mothers, which might affect their access to resources and thus their allocation of maternal behaviour (see Eckardt et al. 2016). Disentangling these factors is no easy endeavour and it was unfortunately not possible in our study, as maternal age and parity highly correlated, and mothers’ previous life experiences were not fully known for all study subjects. Moreover, even when only considering parity, it is not fully clear how this affects the allocation of maternal behaviour. For instance, it is possible that multiparous mothers, through previous pregnancies, become physiologically more mature and/or acquire relevant experience in raising offspring by learning to better interpret and predict their offspring’s needs over time (e.g. Bloomsmith et al. 2003). However, it is also possible that the presence of older siblings, rather than mothers’ higher experience, affects the allocation of maternal resources (e.g. Bard et al. 1997). Siblings, for instance, might not only facilitate contact with other group members, but also provide care to younger offspring as they become more motorically independent, allowing mothers to decrease maternal behaviours more quickly when older siblings are present. Furthermore, it remains unclear whether multiparous mothers are generally more experienced than primiparous mothers, or whether maternal experience may rather increase gradually with each additional offspring. In the future, more longitudinal studies with larger datasets will be needed to understand whether differences in maternal behaviour extend to other behaviours beyond body contact, and if so, whether they truly reflect differences in mothers’ attitude toward their offspring, rather than differences in the offspring’s behaviour and /or in the social context they experience.

Our study had two main limitations. First, it only included captive individuals, which might for instance differ from wild ones by having more resources at their disposal but a more limited choice of social partners—two aspects that might affect both the allocation of maternal behaviour and offspring’s integration into the group. Second, and perhaps most importantly, our study sample was small and might thus have failed to capture important intra-specific variation also within mothers with the same parity (e.g. Bloomsmith et al. 2003). In line with this, our limited sample size did not allow the inclusion of more variables and interactions. Gorilla mothers, for instance, might allocate maternal behaviour differently depending on the sex of the infant’s (Eckardt et al. 2016; Stoinski et al. 2013), and mothers’ experience might specifically affect how they allocate their behaviour through development toward male or female infants. Future studies should ideally include a larger number of individuals from different settings, including wild populations, to capture intra-specific variation and allow for more detailed analyses of maternal behaviours. In this study, for instance, our data only allowed us to model most maternal behaviours as probabilities, rather than proportions, which unfortunately results in a loss of information, as the proportion of time spent in maternal behaviours might be more relevant than their mere occurrence. Moreover, comparisons with mountain gorillas would be especially interesting, as there may be important differences within this genus in terms of maternal behaviour, including how long mothers remain with their sons in the same group (Robbins et al. 2016). In addition, it would be interesting to include other measures of offspring’s integration into the social network and more maternal behaviours, as well as physiological measures of nursing (see Hinde 2009).

Despite these limitations, which clearly limit the generalizability of our findings, we evidenced a limited modulating effect of mothers’ experience on the allocation of maternal behaviour during offspring’s development. Overall, both primiparous and multiparous mothers adjusted their behaviour to the changing needs of their offspring and facilitated their social integration into the group, but multiparous mothers might do it more effectively, at least for some specific behaviours like body contact. Although more studies are needed to understand the mechanisms explaining this parity effect, it is possible that mothers may reduce the costs of maternal behaviour by learning through experience how to optimize its allocation based on the offspring’s changing needs.

## Data Availability

Data will be made available upon reasonable request to the corresponding author.
